# Calcyon mRNA expression in the frontal-striatal circuitry and its relationship to vesicular processes and ADHD

**DOI:** 10.1186/1744-9081-3-33

**Published:** 2007-07-10

**Authors:** Rochellys Diaz Heijtz, Andrey Alexeyenko, F Xavier Castellanos

**Affiliations:** 1 Department of Neuroscience, Karolinska Institutet, Retzius väg 8, Stockholm, 171 77, Sweden; 2 Stockholm Bioinformatics Center, Albanova, Stockholm University, Stockholm, 106 91, Sweden; 3 New York University Child Study Center, 215 Lexington Avenue, New York, New York 10016, USA

## Abstract

**Background:**

Calcyon is a single transmembrane protein predominantly expressed in the brain. Very recently, calcyon has been implicated in clathrin mediated endocytosis, a critical component of synaptic plasticity. At the genetic level, preliminary evidence supports an association between attention-deficit/hyperactivity disorder (ADHD) and polymorphisms in the calcyon gene. As little is known about the potential role of calcyon in ADHD, animal models may provide important insights into this issue.

**Methods:**

We examined calcyon mRNA expression in the frontal-striatal circuitry of three-, five-, and ten-week-old Spontaneously Hypertensive Rats (SHR), the most commonly used animal model of ADHD, and Wistar-Kyoto (WKY; the strain from which SHR were derived). As a complement, we performed a co-expression network analysis using a database of mRNA gene expression profiles of multiple brain regions in order to explore potential functional links of calcyon to other genes.

**Results:**

In all age groups, SHR expressed significantly more calcyon mRNA in the medial prefrontal and orbital frontal cortices than WKY rats. In contrast, in the motor cortex, dorsal striatum and nucleus accumbens, calcyon mRNA expression was only significantly elevated in SHR in younger animals. In both strains, calcyon mRNA levels decreased significantly with age in all regions studied. In the co-expression network analysis, we found a cluster of genes (many of them poorly studied so far) strongly connected to calcyon, which may help elucidate its role in the brain. The pair-wise relations of calcyon with other genes support its involvement in clathrin mediated endocytosis and, potentially, some other membrane/vesicular processes. Interestingly, no link was found between calcyon and the dopamine D1 receptor, which was previously shown to interact with the C-terminal of calcyon.

**Conclusion:**

The results indicate an alteration in calcyon expression within the frontal-striatal circuitry of SHR, especially in areas involved in cognitive processes. These findings extend our understanding of the molecular alterations in SHR, a heuristically useful model of ADHD.

## Background

Attention-deficit/hyperactivity disorder (ADHD) is a common neurobehavioral disorder of childhood onset that can include elements of inattention, hyperactivity and impulsive behavior [1]. The specific aetiology of ADHD is unknown, but family-genetic, twin, adoption, and segregation analyses demonstrate that it is a highly heritable condition [2]. The neurobiology of ADHD is not well understood, but there is converging evidence implicating the catecholamine rich frontal-striatal circuitry [1]. Molecular genetic studies have also focused on hypothesized associations between various catecholamine related genes and ADHD. Several candidate genes have been implicated in ADHD, including genes involved in the dopamine pathway (e.g. DAT1 and dopamine receptors DRD4 and DRD5) [3]. However, the odds ratios for the associations of these genes with ADHD are small (ranging from 1.18 to 1.46), which is consistent with the notion that the genetic vulnerability to ADHD is mediated by multiple genes of small effect.

Recently, an association has been reported between ADHD and a haplotype in the calcyon gene [4]. Calcyon is a single transmembrane protein predominantly expressed in the brain and localized to membranous intracellular compartments within neuronal dendrites and dendritic spines [5]. Studies in non-human primates and rodents have demonstrated that calcyon is highly expressed in multiple brain regions [5-9], including the prefrontal cortex, which mediates cognitive-executive functions (e.g. spatial attention, set-shifting, working memory, and decision-making) [10]. In contrast, in the caudate-putamen (striatum in rodents), calcyon expression is relatively low. Calcyon has been implicated in clathrin mediated endocytosis, a critical component of synaptic plasticity [11]. However, there is limited information about the potential role of the calcyon gene in ADHD. Accordingly, we investigated potential alterations in the expression of calcyon mRNA in the frontal-striatal circuitry of Spontaneously Hypertensive Rats (SHR), the most commonly used animal model of ADHD, and the normotensive Wistar-Kyoto strain (WKY; from which SHR were derived). SHR is the only current animal model that displays all of the behavioural features of ADHD [12,13]. It is relevant to note that hypertension is not present in young SHR but develops gradually from four to twelve weeks of age [14,15]. In order to take into account potential developmental changes, we investigated calcyon mRNA expression at three-, five-, and ten-weeks of age. These ages were selected because they correspond to the prepubertal, adolescent, and adulthood period in humans [16,17]. In addition, we used bioinformatics tools to explore potential calcyon functional connections to other genes.

## Methods

### Animals

Prepubertal (three-week-old), adolescent (five-week-old) and young adult (ten-week-old) male SHR and WKY rats (Charles River Laboratories, Germany) were used. The animals arrived in the laboratory one week before the experiment and were housed in groups of the same strain in standard plastic cages (Type IV Makrolon^®^), under controlled conditions of light: dark cycle (12:12 h, lights on at 07:00 h). Food and water were available ad libitum. The experiments were approved by the Animal Research Ethics Committee of Stockholm and the National Institute of Health Guide on Use of Laboratory Animals.

### Breeding history of animals

SHR (SHR/NCrI) were developed by Okamoto and Aoki at the Kyoto School of Medicine in 1963, from an outbred WKY male with marked elevated blood pressure mated to a female with slightly elevated blood pressure. Brother × sister matings with continued selection for spontaneous hypertension were then transferred to NIH in 1966 from Okamoto at F13, and to Charles River Laboratories from NIH in 1973 at F32 and were caesarean rederived in 1973. WKY (WKY/NCrI) rats originated from outbred Wistar stock transferred from Kyoto School of Medicine to NIH in 1971. This is the same stock from which the SHR strain was developed. They were transferred to Charles River Laboratories in 1974 from NIH, at F11 and caesarean rederived in 1974.

### RNA probe synthesis

Antisense and sense cRNA probes for calcyon were prepared from a 500 base pair Bgl II fragment of rat calcyon cDNA cloned in vector pGEM7zf+ as previously described [8]. The plasmid was linearized with Sac I or EcoRI and then transcribed using T7 (antisense) and SP6 (sense) RNA polymerases, respectively. *In vitro *transcription was carried out using the MAXIscript™ SP6/T7 kit (Applied Biosystems, Sweden) and [α^35^S]-UTP (SJ603, 20 mCi/ml; GE Healthcare, Sweden) according to the manufacture's instructions. The transcripts were purified using NucAway™ Spin Columns (Applied Biosystems, Sweden).

### Hybridization

Expression of calcyon mRNA in the prefrontal cortex and striatum was investigated using *in situ *hybridization technique. Brains were rapidly dissected and frozen on dry ice. Coronal sections (14 μm) of the above areas were prepared on a cryostat and stored at -80°C until use. The *in situ *hybridization was performed as follows. The frozen tissue sections were fixed in cold 4% paraformaldehyde in 0.1 M sodium phosphate buffer pH 7.4 (PBS) for 10 min. After washing with PBS for 5 min, the sections were rinsed in DEPC-H_2_0 (5 min) and deproteinated with 0.1 M HCl for 5 min. The sections were then rinsed twice with PBS (3 min each) and placed into 0.25% acetic anhydride in 0.1 M triethanolamine (pH 8.0) for 20 min at room temperature; washed twice in PBS (3 min each) and dehydrated in 70%, 80% and 100% (2 min each). Sections were air dried and prehybridized [50% deionized formamide (pH 5), 50 mM Tris-HCl, pH 7.6, 25 mM ethylene-diamine-tetraacetate (EDTA), pH 8.0, 20 mM NaCl, 0.25 mg/ml yeast tRNA, 2.5 × Denhardt's solution] for 4 h at 55°C. After draining off the prehybridization buffer, sections were hybridized overnight (14–16 h) in a humidified chamber at 55°C. For hybridization, labeled probes were diluted to a final concentration of 1.0 × 10^6 ^c.p.m./200 μL in a solution containing 50% deionized formamide (pH 5), 0.3 M NaCl, 20 mM Tris-HCl (pH 7.6), 5 mM EDTA (pH 8.0), 10 mM PBS, 0.2 mM dithiothreitol, 0.5 mg/ml yeast tRNA, 0.1 mg/ml poly-A-RNA, 10% dextran sulfate, and 1× Denhardt's solution. After hybridization, the slides were rinsed in 1 × standard saline citrate (SSC), 0.01% SDS (15 min); 1 × SSC, 0.01% SDS (30 min); 50% formamide/0.5 × SSC (1 h); 1 × SSC, and 0.01% SDS (15 min) at 55°C with continuous shaking. The sections were then treated with 1 μg/mL RNase A (Roche, Sweden) in RNase buffer (0.5 M NaCl, 10 mM Tris-HCl, 5 mM EDTA, ph 8.0) for 1 h at 37°C. After two additional washes in 1 × SSC, 0.01% SDS for 30 min, the sections were dehydrated in ascending alcohol series and air dried. Sections were placed against β-Max film (VWR, Sweden) and stored at room temperature for 3 to 5 days. Films were developed in D19 developer for 2 min and in 1:5 dilution of Amfix fixative for 5 min. Non-specific hybridization was determined by incubating sections with the respective ^35^S-UTP-labelled sense cRNA probe for the above cDNA under identical conditions to that of the antisense RNA probe.

### Quantification

Films were scanned with an Epson Perfection 1250 scanner as gray scale film, using 300 pixels and saved as high quality JPEG files. Optical density values were quantified using appropriate software (NIH Image J version 1.29, U.S. National Institutes of Health). A ^14^C step standard (GE Healthcare, Sweden) was included to calibrate optical density readings and convert measured values into nCi/g. Optical density measurements were averaged from two adjacent sections per animal and region of interest for statistical analyses. All comparisons between groups were made on sections hybridized together, under identical conditions and exposed for the same periods of time to β-Max film (VWR, Sweden).

### Criteria used for evaluation of brain regions

Anatomical regions were identified and subdivided for densitometric analysis according to the stereotaxic atlas of Paxinos and Watson [18]. The rat prefrontal cortex consists of two spatially separated areas; namely, the medial and orbital regions [19]. The medial prefrontal cortex can be divided into infralimbic (IL), prelimbic (PrL), dorsal and ventral anterior cingulate, and medial precentral cortical area (PrCm). The orbital frontal cortex can be divided into medial, ventral and lateral orbital cortices, and agranular insular cortex. Our measurements of the orbital frontal cortex contained both the ventral and lateral orbital cortices, and for the medial prefrontal cortex contained the IL, PrL and cingulate (approximately + 2.6 mm posterior to bregma). The measurements of the motor cortex were taken from primary motor cortex (M1) (approximately +1.6 mm posterior to bregma). The measurements of the nucleus accumbens were taken from the shell region (approximately +1.6 mm posterior to bregma). The measurements from the rostral, middle, and caudal striatum were taken at approximately +1.6, +0.7, and -0.4 mm posterior to bregma, respectively. For further details see [20].

### Statistical analysis of calcyon mRNA expression

Statistical analysis of mRNA expression was performed using factorial ANOVA (STRAIN and AGE as main factors; each STRAIN × AGE group contained 5 animals as replicates) for each brain region. The pair-wise post-hoc comparisons were made using the Bonferroni/Dunn test. For all analyses, significance was assigned at the P < 0.05 level. All data are presented as means ± S.E.M.

### Co-expression network analysis

The dataset from the Mouse Atlas of Gene Expression [21] was downloaded from Gene Expression Omnibus as GEO Series #4726. For each gene (a total of 11,328 genes from ENSEMBL mouse genome version) in each sample, its relative expression level was used, i.e. a log-transformed ratio of the SAGE tag abundance in the sample to its average abundance across all the samples. This normalization was needed to compute Pearson linear correlation coefficients. The correlation coefficients between mRNA expression profiles were computed across developing and adult brain tissue samples (missing observations excluded pair-wise). For comparison, alternative co-expression values were calculated identically across *all *the tissues of the Atlas. Pearson coefficients exceeding r > 0.55 (P < 0.001) for mRNA expression correlations were used to link genes with the tightest connections to each other or to calcyon. However, any two given genes may not show direct links in such a network. Indeed, even if A <-> B and B <-> C pairs are strongly correlated, A <-> C may be more weakly correlated and thus not discernable. Correlations between calcyon and genes of particular interest were calculated for each of the Mouse Atlas genes. The genes represented as color-marked nodes (see results section) are: dopamine receptors, clathrin chains, ADHD-associated genes from OMIM database, and a group of genes we found co-expressed with calcyon as human orthologs (found at InParanoid resource, [22]) in the Human Tissue Atlas ([23]; correlation values not shown). In total, more than 900,000 gene pairs were analyzed for functional linkage. Hence, even if a direct connection were not visible, it could be discovered via neighbors that share genes in the network.

A number of genes did not have valid data in the Mouse Atlas (e.g., the two clathrin light chains and dopamine receptors 3, 4, and 5). Some functional links may also exist but did not exceed the threshold we selected (r > 0.55) for determining the presence of mRNA co-expression. As has been shown, one of a pair of interacting genes can be expressed permanently but the joint activity can be regulated by transient expression of the other gene [24]. Such protein pairs would not be detected in our co-expression network analyses. Finally, the absence of some genes in sub-networks may be explained by low sensitivity (i.e., only a minority of functionally related gene pairs can usually be found by means of pure mRNA co-expression).

All mouse genes used in the co-expression network analysis are spelled according to the Mouse Genome Informatics (MGI) data base. For example, the mouse dopamine D1 receptor is abbreviated as Drd1a, while the human or rat dopamine D1 receptor is abbreviated as DRD1. Images were prepared with the network visualization tool Medusa [25].

## Results

The specificity of the signals obtained with *in situ *hybridization was confirmed using a sense probe. Background levels were very low in all sections analyzed (data not shown).

### Medial prefrontal cortex

**Figure 1:**
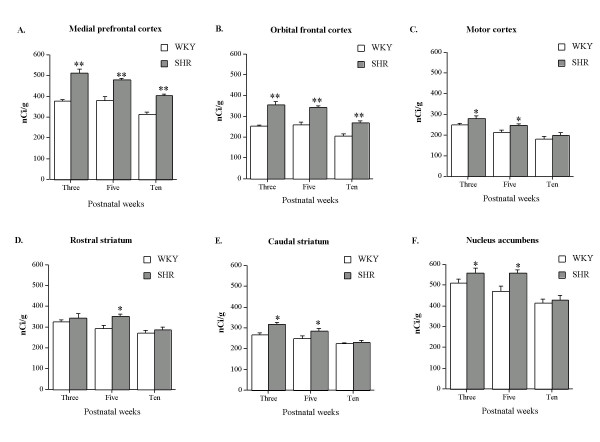
Expression of calcyon mRNA in the frontal-striatal circuitry of SHR and WKY rats during various postnatal ages. Values are shown as means ± S.E.M., n = 5 per each group. **P < 0.0001; * P < 0.05 compared to WKY rats of the same age. For further details see the results section.

**Figure 2:**
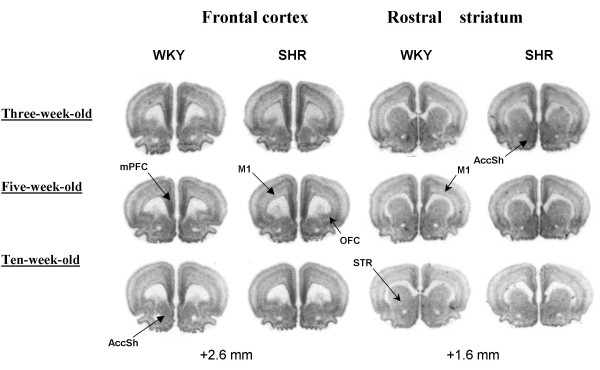
Representative autoradiograms showing calcyon mRNA at the level of the frontal cortex and rostral striatum in three-, five-, and ten-week-old SHR and WKY rats. Coronal sections are from approximate bregma levels +2.6 and +1.6, respectively (arbitrary scale). The abbreviations are as follows: mPFC, medial prefrontal cortex; OFC, orbital frontal cortex; M1, primary motor cortex; STR, striatum; AccSh, nucleus accumbens (shell region).

All regional analyses consisted of two-way factorial ANOVAs with main factors STRAIN, DF = [1,24] and AGE, DF = [2,24] and STRAIN × AGE, DF = [2,24]. ANOVA of the medial prefrontal cortex revealed a significant main effect of STRAIN [F = 130.3; P < 0.0001] and AGE [F = 32.9; P < 0.0001], but failed to reveal a STRAIN × AGE interaction. Post-hoc analysis with the Bonferroni/Dunn test showed that the three-, five- and ten-week old SHR expressed significantly (P < 0.0001) higher levels of calcyon mRNA when compared to WKY rats of the same age (see Figs. 1A and 2). In both strain of rats, the three-, and five-week old rats expressed significantly (P < 0.05) higher levels of calcyon mRNA than the ten-week-old rats.

### Orbital frontal cortex

ANOVA of the orbital cortex revealed a significant main effect of STRAIN [F = 126.6; P < 0.0001] and AGE [F = 34.9; P < 0.0001], but failed to reveal a STRAIN × AGE interaction. Similar to the medial prefrontal cortex, the three-, five- and ten-week old SHR were found to express significantly (P < 0.0001) higher levels of calcyon mRNA when compared to WKY rats of the same age (see Figs. 1B and 2). Moreover, in both strain of rats, calcyon mRNA expression was significantly (P < 0.0001) higher in the three-, and five-week-old rats than in the ten-week old rats.

### Motor cortex

ANOVA of the primary motor cortex revealed a significant main effect of STRAIN [F = 10.0; P = 0.004] and AGE [F = 25.9; P < 0.0001], but failed to reveal a STRAIN × AGE interaction. Post-hoc analysis showed that the three-, and five-week-old SHR expressed a rather small but significant (P < 0.05) increase in calcyon mRNA when compared to the WKY rats of the same age (see Figs. 1C and 2). In both strains of rats, the three-, and five-week-old rats expressed significantly (P < 0.05) higher levels of calcyon mRNA than the ten-week-old rats.

### Striatum

ANOVA of the rostral, middle, and caudal striatum revealed a significant main effect of STRAIN ([F = 8.2; P = 0.009], [F = 8.4; P = 0.008], and [F = 14.3; P = 0.001], respectively) and AGE ([F = 9.5; P = 0.001], [F = 23.2; P < 0.0001], and [F = 21.4; P < 0.001], respectively), but failed to reveal a STRAIN × AGE interaction. In both the rostral and middle striatum, post-hoc analysis showed that the five-week-old SHR expressed a rather small but significant (P < 0.05) increase in calcyon mRNA when compared to WKY rats of the same age (see Figs. 1D and 2). In addition, in both strain of rats the three-week-old rats expressed significantly (P < 0.05) higher calcyon mRNA levels than the ten-week-old rats. In the caudal striatum, post-hoc analysis showed that the three-, and five-week-old SHR expressed a rather small but significant (P < 0.05) increase in calcyon mRNA when compared to WKY rats of the same age (see Figs. 1E and 2). In both strains of rats the three-, and five-week-old rats expressed significantly (P < 0.05) higher calcyon mRNA levels than the ten-week-old rats.

### Nucleus accumbens

ANOVA of the nucleus accumbens (shell region) revealed a significant main effect of STRAIN [F = 9.3; P = 0.005] and AGE [F = 17.9; P < 0.0001], but failed to reveal a STRAIN × AGE interaction. Post-hoc analysis showed that the three-, and five-week-old SHR expressed a rather small but significant (P < 0.05) increase in calcyon mRNA when compared to WKY rats of the same age (see Figs. 1F and 2). In addition, in both strain of rats the three-, and five-week-old rats expressed significantly (P < 0.05) higher calcyon mRNA levels than the ten-week-old rats.

### Co-expression network analysis

In order to shed more light on calcyon function, we explored its functional connections to other genes by performing network analyses. We reasoned that the most informative pattern would be revealed by analyzing a co-expression network in brain tissue. The Mouse Atlas of Gene Expression [21] was suitable for this purpose, as it contains the expression patterns of 11,328 genes. For a confident co-expression analysis, one should use datasets in which most of the genes have been observed in multiple expression conditions. We were aware of 13 large expression sets in human, mouse, and rat. Calcyon is a relatively novel and poorly studied gene. Thus apart from the Mouse Tissue Atlas, only one Human Atlas ([23]; considered in the paper as well) had a calcyon expression profile – but this atlas did not have enough various brain tissue conditions.

**Figure 3:**
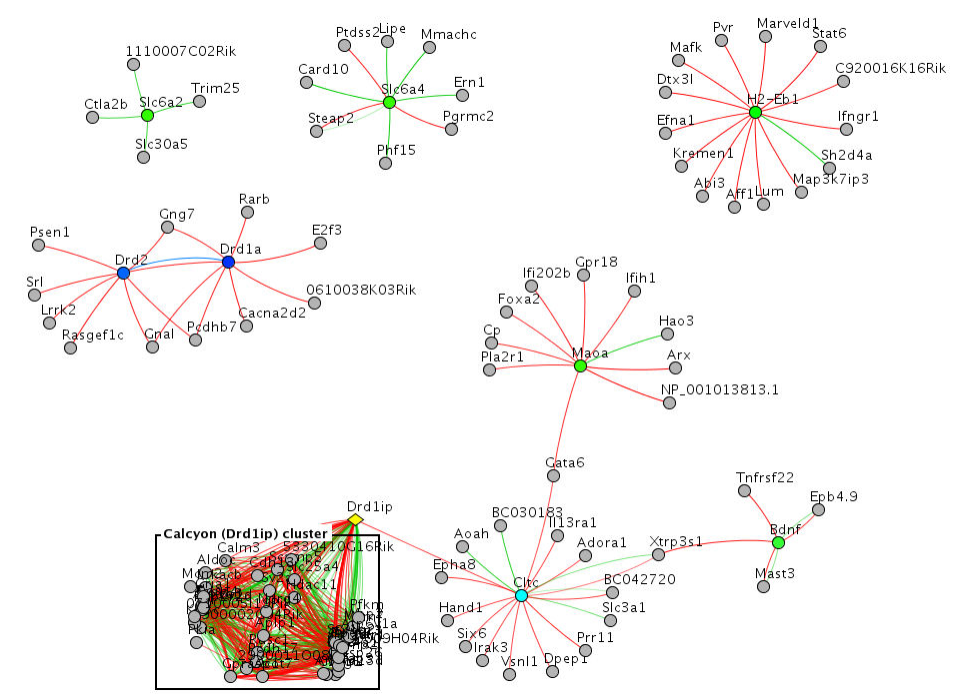
Co-expression network of calcyon in the developing and adult mouse brain are shown. Red lines: positive co-expression (Pearson r > 0.55, P < 0.001) in brain tissue; Green lines: positive co-expression (Pearson r > 0.55, P < 0.001) across all tissues of the Mouse Atlas; Blue lines: paralogs; Nodes: genes; Yellow: calcyon; Blue: dopamine receptors; Cyan: clathrin subunit (only data for the heavy chain gene was available); Green: ADHD-related genes (by OMIM annotation); Box: the network cluster of strong positive relation to calcyon (see more details in Fig. 4 and Table 1).

**Figure 4:**
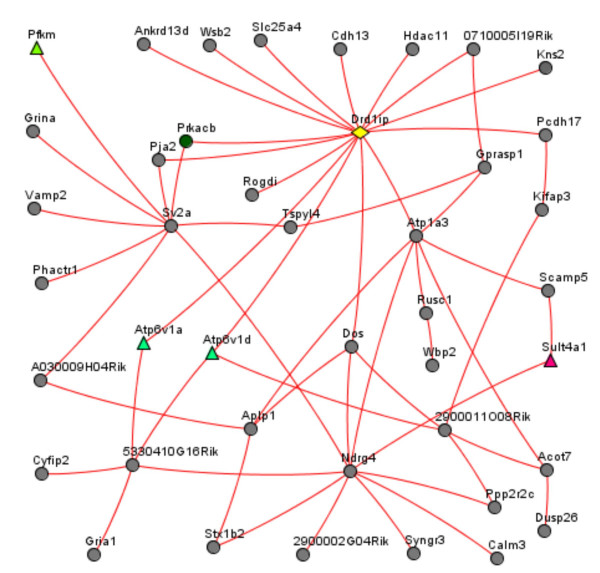
Cluster of genes of high positive co-expression with calcyon in the mouse brain. Each of the genes shown had a Pearson correlation coefficient of mRNA profiles with calcyon exceeding r > 0.55 (P < 0.001). Because of the high number of such connections between genes (~8 links per gene), the ARACNE algorithm [46] was applied to reduce connectivity straightforwardly. Namely, for each triangle (genes i, j, and k connected to each other) the algorithm removed the weakest 3 links; thus the number of links was reduced 6-fold while all the linked genes were retained. Note that even though some genes have a distant path to calcyon (1 or 2 nodes away), each had a Pearson correlation value with calcyon r > 0.55. Nodes (genes); Yellow diamond (Calcyon); Color triangles (Members of known metabolic pathways); Grey circles (Other genes producing a tight cluster of mutually co-expressed genes around calcyon). For further details about the annotations see Table 1.

While analyzing functional links in the co-expression network, special attention was paid to genes previously suggested as being related to calcyon. Figure 3 shows the calcyon network and the potentially related genes in the developing and adult mouse brain tissues. No significant links were found between calcyon and dopamine receptors Drd2 and Drd1a (the latter previously having been shown to interact with calcyon), even when indirect connections via other genes were considered. The dopamine receptors Drd3, Drd4, and Drd5 were not present in the database. Several ADHD-associated genes with available mRNA profiles did not relate to calcyon either. However, multiple, and mostly strong, connections were revealed between calcyon and genes whose annotations suggested that they are involved in synaptic plasticity, endocytosis and/or vesicle formation (e.g. clathrin heavy chain, and the ionotropic glutamate receptor, AMPA1) (see Fig. 4 and Table 1). A number of genes in this calcyon-related cluster were novel, as indicated by absence of any annotation or gene names (0710005I19Rik, 2900002G04Rik, 2900011O08Rik, 5330410G16Rik, A030009H04Rik); others were only sparsely documented (Aplp1, Pja2, and Rusc1).

**Table 1:** Genes of the calcyon-related network cluster. Gene name, ENSEMBL gene ID, and ENSEMBL gene description are provided from left to right.

***Gene***	***ENSEMBL ID***	***ENSEMBL description***
Acot7	ENSMUSG00000028937	acyl-CoA thioesterase 7 [Acc:MGI:1917275]
Ankrd13d	ENSMUSG00000005986	ankyrin repeat domain 13 family, member D [Acc:MGI:1915673]
Aplp1	ENSMUSG00000006651	amyloid beta (A4) precursor-like protein 1 [Acc:MGI:88046]
Atp1a3	ENSMUSG00000040907	ATPase, Na+/K+ transporting, alpha 3 polypeptide [Acc:MGI:88107]
Atp6v1a	ENSMUSG00000052459	ATPase, H+ transporting, lysosomal V1 subunit A [Acc:MGI:1201780]
Atp6v1d	ENSMUSG00000021114	ATPase, H+ transporting, lysosomal V1 subunit D [Acc:MGI:1921084]
Calm3	ENSMUSG00000019370	calmodulin 3 [Acc:MGI:103249]
Cdh13	ENSMUSG00000031841	cadherin 13 [Acc:MGI:99551]
Cyfip2	ENSMUSG00000020340	cytoplasmic FMR1 interacting protein 2 [Acc:MGI:1924134]
Dos	ENSMUSG00000035640	downstream of Stk11 [Acc:MGI:1354170]
Dusp26	ENSMUSG00000039661	dual specificity phosphatase 26 (putative) [Acc:MGI:1914209]
Gprasp1	ENSMUSG00000043384	G protein-coupled receptor associated sorting protein 1 [Acc:MGI:1917418]
Gria1	ENSMUSG00000020524	glutamate receptor, ionotropic, AMPA1 (alpha 1) [Acc:MGI:95808]
Grina	ENSMUSG00000022564	glutamate receptor, ionotropic, N-methyl D-asparate-associated protein 1 [Acc:MGI:1913418]
Hdac11	ENSMUSG00000034245	histone deacetylase 11 [Acc:MGI:2385252]
Kifap3	ENSMUSG00000026585	kinesin-associated protein 3 [Acc:MGI:107566]
Kns2	ENSMUSG00000021288	kinesin 2 [Acc:MGI:107978]
Ndrg4	ENSMUSG00000036564	N-myc downstream regulated gene 4 [Acc:MGI:2384590]
Pcdh17	ENSMUSG00000035566	protocadherin 17 [Acc:MGI:2684924]
Pfkm	ENSMUSG00000033065	phosphofructokinase, muscle [Acc:MGI:97548]
Phactr1	ENSMUSG00000054728	phosphatase and actin regulator 1 [Acc:MGI:2659021]
Pja2	ENSMUSG00000024083	praja 2, RING-H2 motif containing [Acc:MGI:2159342]
Ppp2r2c	ENSMUSG00000029120	protein phosphatase 2 (formerly 2A), regulatory subunit B (PR 52), gamma isoform [Acc:MGI:2442660]
Prkacb	ENSMUSG00000005034	protein kinase, cAMP dependent, catalytic, beta [Acc:MGI:97594]
Rogdi	ENSMUSG00000022540	rogdi homolog (Drosophila) [Acc:MGI:1913299]
Rusc1	ENSMUSG00000041263	RUN and SH3 domain containing 1 [Acc:MGI:1919546]
Scamp5	ENSMUSG00000040722	secretory carrier membrane protein 5 [Acc:MGI:1928948]
Slc25a4	ENSMUSG00000031633	solute carrier family 25 (mitochondrial carrier, adenine nucleotide translocator), member 4 [Acc:MGI:1353495]
Stx1b2	ENSMUSG00000030806	syntaxin 1B2 [Acc:MGI:1930705]
Sult4a1	ENSMUSG00000018865	sulfotransferase family 4A, member 1 [Acc:MGI:1888971]
Sv2a	ENSMUSG00000038486	synaptic vesicle glycoprotein 2 a [Acc:MGI:1927139]
Syngr3	ENSMUSG00000007021	synaptogyrin 3 [Acc:MGI:1341881]
Tspyl4	ENSMUSG00000039485	TSPY-like 4 [Acc:MGI:106393]
Vamp2	ENSMUSG00000020894	vesicle-associated membrane protein 2 [Acc:MGI:1313277]
Wbp2	ENSMUSG00000034341	WW domain binding protein 2 [Acc:MGI:104709]
Wsb2	ENSMUSG00000029364	WD repeat and SOCS box-containing 2 [Acc:MGI:2144041]
0710005I19Rik	ENSMUSG00000041141	RIKEN cDNA 0710005I19 gene [Acc:MGI:1918941]
2900002G04Rik	ENSMUSG00000041020	RIKEN cDNA 2900002G04 gene [Acc:MGI:1917474]
2900011O08Rik	ENSMUSG00000044117	RIKEN cDNA 2900011O08 gene [Acc:MGI:1914504]
5330410G16Rik	ENSMUSG00000035964	RIKEN cDNA 5330410G16 gene [Acc:MGI:1915187]
A030009H04Rik	ENSMUSG00000043419	RIKEN cDNA A030009H04 gene [Acc:MGI:1915359]

## Discussion

The present study demonstrates for the first time that the transcript encoding calcyon is upregulated in the frontal-striatal circuitry of SHR when compared to WKY rats, with the strongest strain differences found in the youngest animals in motor cortex, dorsal caudate, and nucleus accumbens. By contrast, in the medial prefrontal, and orbital frontal cortices increased transcript expression of calcyon was observed throughout the different developmental stages investigated (prepubertal, adolescence, and adulthood). Taken together with recent findings (see below), the present results indicate a potential alteration in clathrin mediated endocytosis and synaptic plasticity in the frontal-striatal circuitry of SHR involved in motor and cognitive functions.

The expression of calcyon mRNA in frontal-striatal circuitry has been previously described in adult Sprague-Dawley rats [8]. Calcyon was found to be highly expressed in the medial prefrontal cortex, with low to moderate expression throughout the dorsal striatum and nucleus accumbens. In the present study, we also found a similar pattern of expression in both SHR and WKY rats, which is similar to that seen in primates [5,7]. Interestingly, calcyon mRNA expression was consistently higher in prepubertal rats (three-week-old) compared to young adult rats (ten-week-old) in both strains. We have also found a similar developmental pattern in Sprague-Dawley rats (Diaz Heijtz, unpublished results). These observations of a developmental gradient may also be relevant for understanding the neurobiology of schizophrenia, in which several studies have found calcyon to be upregulated in dorsolateral prefrontal cortex in postmortem brain tissue from patients with schizophrenia [26-29]. Moreover, these studies suggest the speculation that elevated levels of calcyon in patients with schizophrenia may result from an altered developmental program of synaptic plasticity.

Support for a role of calcyon in the aetiology of ADHD comes primarily from genetic studies. In a recent genome-wide linkage scan study for loci influencing ADHD, the calcyon gene was found to coincide with one of the highest positive linkage sites identified at chromosome 10q26 [30]. Some patients with terminal or interstitial deletions involving chromosome bands 10q25.2–26 have a characteristic phenotype, which may include learning difficulties, aggression and hyperactivity [31]. More recently, the inheritance of nine polymorphisms in the calcyon gene was examined in a large clinically referred sample of affected children with ADHD and their immediate families using the transmission-disequilibrium test. This study reported evidence for excess transmission of the most common calcyon haplotype, designed C1 [4]. In addition, this haplotype was positively associated with both the hyperactive/impulsive and inattentive symptoms of ADHD, supporting the idea that variations in calcyon may contribute to both deficits in motor control and cognitive functions of the disorder. This notion is indirectly supported by the result of the present study demonstrating alterations in calcyon mRNA expression in subregions of the prefrontal cortex and striatum of SHR, which are involved in motor control and cognitive-executive functions. However, the finding that the expression of calcyon mRNA is unaltered in the motor cortex, dorsal striatum and nucleus accumbens of young adult SHR suggests that calcyon is more likely to contribute to deficits in motor control during early development.

Previous studies have suggested that calcyon functions as a dopamine D1 receptor interacting protein (DRD1IP) enabling the typically Gs-linked dopamine D1 receptor (DRD1) to stimulate intracellular calcium release, after initial activation of a heterologous G_q_-linked G-protein coupled receptor [5,32,33]. However, several authors observed the presence of high levels of calcyon mRNA expression in brain regions not associated with DRD1 [7,8]. A substantial proportion of this mismatch was proposed to be related to a potential interaction of calcyon with DRD5, which contain a region similar in sequence to the core calcyon binding domain of DRD1. New evidence indicates a role for calcyon in clathrin mediated endocytosis in the brain. Clathrin-coated vesicle assembly and disassembly are known to be regulated by multiple adaptor and accessory proteins, most of which are ubiquitous and interact with clathrin heavy chain [34]. Using two-hybrid screen systems, the cytosolic domain of calcyon was shown to interact with the heavy chain binding domain and C-terminal regions of the light chain [11]. Moreover, the addition of a fusion protein containing the calcyon C-terminus stimulated clathrin self-assembly *in vitro *[11].

The results of the co-expression network analysis in mouse support calcyon involvement in clathrin mediated endocytosis. Indeed, clathrin, ionotropic glutamate receptors, vesicle proteins Vamp2 and Sv2a, secretory carrier protein Scamp 5 (see the complete list in Table 1) were directly connected to calcyon. This set of genes has been found to co-express with calcyon also in the human brain (data not shown). Previous genetic studies have implicated several genes involved in the vesicular release of neurotransmitters (e.g. SNAP-25) in ADHD [35-40]. We also observed that SNAP-25 strongly co-expressed with calcyon in human tissues, but its mouse ortholog data was unavailable. Interestingly, no significant links were found between calcyon and Drd1a, which has been previously shown to interact with the C-terminal of calcyon [5].

The above information is consistent with anatomical findings localizing calcyon to vesicular compartments in dendritic spines and axon terminals, two sites in the brain where clathrin mediated endocytosis is essential for efficient synaptic neurotransmission and plasticity associated with learning and memory [41,42]. For example, clathrin mediated endocytosis plays a crucial role in the stimulus dependent removal of alpha-amino-3-hydroxy-5 methylisoxazole-4-propionic acid (AMPA) receptors from synapses in hippocampal dendritic spines during the synaptic weakening phenomenon of long-term depression (LTD) [43]. Preliminary studies have found that LTD is attenuated in hippocampal neurons from calcyon knock-out mice, but enhanced in neurons from calcyon over-expressing mice [44]. Interestingly, there is also evidence indicating elevated AMPA receptor function in the prefrontal cortex of SHR when compared to WKY rats [45].

Calcyon over-expressing mice have also been tested in some behavioural tasks commonly associated with schizophrenia pathology. Compared to wild type mice, calcyon over-expressing mice appear to have elevated basal locomotor activity, increased exploratory behaviours in an elevated plus maze, and impaired prepulse inhibition [44]. Further investigations using the calcyon knock-out and over-expressing mice might provide additional mechanistic insights regarding the potential role of calcyon for regulating synaptic plasticity at excitatory synapses, and how it may relate to behavioural alterations.

## Conclusion

In this study we analyzed the expression of calcyon mRNA in the frontal-striatal circuitry of SHR and WKY rats at different postnatal ages. In addition, we performed a co-expression network analysis using a database of mRNA gene expression profiles of multiple brain regions in order to explore potential functional links of calcyon to other genes. We found calcyon to be upregulated in various subregions of the prefrontal cortex and striatum of SHR when compared to WKY rats. Importantly, these alterations were influenced by age. Results from the co-expression network analysis support the notion that calcyon may be involved in vesicular processes. We speculate that elevated levels of calcyon might produce both cognitive and motor dysfunction in an age-dependent manner in patients with ADHD via effects of synaptic plasticity (e.g. involving receptor endocytosis).

## Competing interests

The author(s) declare that they have no competing interests.

## Authors' contributions

RDH designed and carried out the study, conducted statistical analyses, and drafted the manuscript. AA performed the co-expression analysis and helped draft the manuscript. FXC participated in the overall study design and helped draft the manuscript. All authors read and approved the final manuscript.
